# Improving Retention in Care Among Pregnant Women and Mothers Living With HIV: Lessons From INSPIRE and Implications for Future WHO Guidance and Monitoring

**DOI:** 10.1097/QAI.0000000000001366

**Published:** 2017-05-15

**Authors:** Nigel C. Rollins, Shaffiq M. Essajee, Nita Bellare, Meg Doherty, Gottfried O. Hirnschall

**Affiliations:** * Department of Maternal, Newborn, Child and Adolescent Health, World Health Organization, Geneva, Switzerland; and; † Department of HIV/AIDS, World Health Organization, Geneva, Switzerland.

**Keywords:** implementation research, PMTCT, retention in care, HIV, Malawi, Nigeria, Zimbabwe

## Abstract

Identifying women living with HIV, initiating them on lifelong antiretroviral treatment (ART), and retaining them in care are among the important challenges facing this generation of health care managers and public health researchers. Implementation research attempts to solve a wide range of implementation problems by trying to understand and work within real-world conditions to find solutions that have a measureable impact on the outcomes of interest. Implementation research is distinct from clinical research in many ways yet demands similar standards of conceptual thinking and discipline to generate robust evidence that can be, to some extent, generalized to inform policy and service delivery. In 2011, the World Health Organization (WHO), with funding from Global Affairs Canada, began support to 6 implementation research projects in Malawi, Nigeria, and Zimbabwe. All focused on evaluating approaches for improving rates of retention in care among pregnant women and mothers living with HIV and ensuring their continuation of ART. This reflected the priority given by ministries of health, program implementers, and researchers in each country to the importance of women living with HIV returning to health facilities for routine care, adherence to ART, and improved health outcomes. Five of the studies were cluster randomized controlled trials, and 1 adopted a matched cohort design. Here, we summarize some of the main findings and key lessons learned. We also consider some of the broader implications, remaining knowledge gaps, and how implementation research is integral to, and essential for, global guideline development and to inform HIV/AIDS strategies.

## INTRODUCTION

The development of antiretroviral treatment (ART) that effectively suppresses HIV viral load has fundamentally transformed the tragedy that HIV/AIDS has represented for mothers and children living in high prevalence settings. Equally important have been the political efforts to ensure its affordability and distribution. Challenges remain, however, that compromise the benefit that ART can yield for these and other populations: access to accurate information on ART and the opportunity to enforce their health care preferences obstruct many women from gaining effective treatment; unreliable health systems that fail to deliver consistent, quality services or neglect the rights and needs of women and girls undermine trust and confidence in health care workers; social determinants and stigma profoundly affect the decisions of women whether to return to local health facilities or not, regardless if treatment is beneficial.

Identifying women living with HIV, initiating them on ART, and retaining them and their children in care are among the challenges facing the current generation of health care managers and public health researchers. Unless the proportions of affected pregnant women and mothers identified, initiated, and retained in care are in excess of 90%, the UNAIDS Super-Fast-Track Target of reducing the number of children newly infected to less than 20,000 by 2020^1^ is no more than a commendable aspiration. The question is, now that the low hanging fruit of reaching the most accessible populations of pregnant women with effective ART has been harvested, how do national health authorities together with the global health community address the remaining gaps?

Implementation research (IR) attempts to solve a wide range of implementation problems by trying to understand and work within real-world conditions to find solutions that carry an intention to its effect.^2^ It is distinct from clinical research in many ways yet demands similar standards of conceptual thinking and discipline to generate robust evidence that can, to some extent, be generalized to inform policy and service delivery. The importance of IR is increasingly recognized, and some scientific journals including *JAIDS* are dedicating sections to reporting findings of IR studies.^4^

In 2010, national departments of health and World Health Organization (WHO) convened local research organizations and key partners in Malawi, Nigeria, and Zimbabwe to participate in IR prioritization exercises. Key challenges for maximizing the effectiveness of HIV/AIDS interventions were reviewed and research questions identified.^5^ Following public calls for proposals, 6 IR projects were funded by Global Affairs Canada: all focused on testing approaches for improving rates of retention in care among pregnant women and mothers living with HIV and ensuring their continuation of ART. This reflected the priority given by ministries of health, program implementers, and researchers in each country to the importance of women living with HIV returning to health facilities for routine care, adherence to ART, and improved health outcomes.

Here, we summarize some of the main findings and key lessons learned. We also consider some of the broader implications, remaining knowledge gaps, and how WHO emphasizes the contribution of IR to global guideline development and HIV/AIDS strategies. As such, IR can help to optimize the benefits of ARV and other essential maternal, newborn, and child health (MNCH) interventions.

## THE STUDIES

Integrating and Scaling up PMTCT through Implementation Research (INSPIRE) included 2 IR studies each in Malawi, Nigeria, and Zimbabwe. PIs were all national scientists based in local institutions. Study sites were principally rural or semirural, and all studies were implemented within the context of routine health systems that were either already providing lifelong ART to all pregnant women and lactating mothers (Option B+) or established lifelong ART as part of the project set-up. Five of the studies were cluster randomized controlled studies; however, because of programmatic reasons, 1 study was unable to independently randomize and reverted to a matched cohort design. None deployed more professional health staff to work at the front line of service delivery. All used routine health systems' data but supplemented them with additional data inputs. Approximately 5100 women living with HIV were enrolled across all the studies.

Three studies examined ways to engage women already living with HIV to provide expert support to other pregnant women or mothers who had recently learned their HIV status and were starting ART. Three studies tested interventions that principally focused on improving the quality of health service delivery.

## PEER SUPPORT INTERVENTIONS AND RETENTION IN CARE AND OTHER HIV-RELATED OUTCOMES

In Malawi, the PMTCT Uptake and REtention (PURE) study showed that mothers living with HIV who received expert peer support were significantly more likely to be retained in care at 24 months postinitiation of ART; this was true regardless if the support was provided at facilities or in the community (approximately 80%, 83% vs. 66%).^6^ The positive effect of peer support was primarily seen in the period between 12 and 24 months after ART initiation. Rates of attrition were between 30% and 40% less among women receiving expert peer support. The number of women coming back into care after a period of defaulting was also higher in settings where there was community-based expert peer support compared with either standard of care or facility-based expert peer support. At 6 months after ART initiation, 84% of women living with HIV were virally suppressed; among those with viral load >1000 copies/mL and successful amplification for resistance testing, confirmed HIV resistance was detected in 35%. Women without any default episodes were more likely to have achieved virological suppression.

In Nigeria, the Mother Mentor (MoMent) study demonstrated how mothers who received structured peer support from trained, supervised mentor mothers were much more likely to remain in care at 6 months postpartum compared with women receiving standard of care (62% vs. 25%).^7^ This difference was present despite control sites also offering some individual peer support through local women living with HIV who were contracted, through the routine services, to provide similar help and counseling. The difference in effect appeared because of the quality of training and regular supervision provided to the expert mentor mothers and was not related to the ratio of women to local peer supporter or expert mentor mother. The positive impact of expert mentor mothers was also associated with higher overall clinic attendance even when full retention was not achieved. At 6 months postpartum, 58% of all mothers on ART were virally suppressed. In multivariate analyses, women receiving expert mentor mother support were nearly 5 times more likely to be virally suppressed than mothers receiving standard of care support. Infants of mothers supported by expert mentor mothers were significantly more likely to present for testing of HIV status between 35 and 62 days of life (64% vs. 35%). Less than 1% of HIV-exposed infants were HIV infected at this time.

The EPAZ study in Zimbabwe, reported that coordinating mother support groups delivering a structured syllabus of HIV-related information and other support, was not associated with a statistically significant difference in rates of retention of infants born to mothers living with HIV at 12 months of age: 69% of infants born to mothers in the intervention arm were retained in care at 12 months compared with 61% in the control arm.^8^ A range of secondary outcomes including mothers' attendance at 12 months (71% vs. 61%), regularity of clinic attendances (78% vs. 71%), infant HIV testing rates (72% vs. 62%) were consistently higher, in the intervention versus control sites. The authors suggested that selective enrollment of “low-risk” mothers may have accounted for the lack of detectable difference in infant retention rates. About a quarter of eligible women did not consent to participate possibly because of the need to obtain partner agreement. The support groups were led by unpaid volunteers and did not provide transport or other material incentives or engage in economic or livelihood-strengthening activities—thought by the research team not to be sustainable within public health facilities in Zimbabwe.

In a joint analysis of the 3 studies testing peer support interventions, the option of expert mothers providing an expanded range of services and sharing health care professionals' workload was viewed positively by prevention of mother-to-child transmission (PMTCT) clients and health workers alike.^9^ As the roles of expert mothers evolved rapidly during the research period, formal training and certification, structured supervision, and standardized remuneration were identified as key elements for successful peer support interventions in similar settings. None of the 3 studies identified protection of client or expert mother confidentiality to be a significant barrier to scale up of this type of intervention in PMTCT programs.

## HEALTH SYSTEMS' INTERVENTION AND RETENTION IN CARE AND OTHER HIV-RELATED OUTCOMES

Three studies tested interventions directed at modifying health service delivery or adapting specific elements of care. However, none reported improved rates of retention among mothers living with HIV during the 12-month postpartum.

The Promoting Retention among Infants and Mothers Effectively (PRIME) study in Malawi sought to learn whether an integrated service delivery approach in which pregnant women and mothers could access all relevant services, both antenatal and postnatal, at the one time would improve retention in care at 12 months postpartum. In addition, an SMS messaging intervention was introduced to fast-track community volunteers' responses and tracing of women who did not attend clinic appointments.^10^ Neither additional clinic staff nor financial incentives were offered as part of the intervention package. Based on a very strict study definition—attendance at 12 months postpartum and also attendance at every scheduled visit before then (±14 days)—rates of maternal retention at 12 months postpartum were only between 19% and 25% in any arm. When the same data were analyzed using the national definition for retention in care—alive and on ART without any gap in care >60 days after a scheduled visit—this equated to rates between 67% and 72%. Despite enormous efforts by the study team to constructively and consistently support intervention sites, changes in facility organization and practices to deliver an integrated mother-infant package seemed out of reach. Over the course of the study, however, mothers and infants increasingly attended the integrated clinics, suggesting that health systems' interventions need much more time to deploy and effect change compared with more versatile and adaptable interventions such as expert peer support. High rates of loss to follow-up were also reported among pregnant women before delivery, highlighting the need for interventions directed at this specific population.

Lafiyan Jikin Mata (Excellent Health for Mothers) in Nigeria tested a continuous quality improvement (CQI) intervention to mitigate what local health workers and managers identified as drivers of poor retention in care, ie, long waiting times and poor quality of service at clinics. Rates of retention were no different by study arm regardless of the definition used.^11^ Again, the study team invested enormous effort and commitment to support local health facility teams to learn and implement CQI and the Breakthrough Series methods.^12^ Facility teams enthusiastically engaged in the CQI process and actively tested change ideas—more than 41 in total across the intervention sites. Weaknesses in the theory of change, insufficient time to properly implement the intervention, civil strife, and disruptions in the health system may all have contributed to the lack of impact on retention in care. Rates of infant HIV testing around 6 weeks of age were, however, significantly higher in intervention sites compared with the control arm.

The Evidence for Elimination study (E4E) in Zimbabwe tested whether point-of-care (POC) testing and CD4 count–specific counseling at the time of ART initiation would further motivate women to remain in care. Although CD4 testing is not required for initiating ART among pregnant and breastfeeding women in Zimbabwe, it is used for monitoring ART. In the intervention arm, CD4 testing was conducted with POC devices while blood samples were sent to a centralized laboratory in the standard of care arm. Approximately 50% of women were retained in care 12 months postinitiation in both arms.^13^ POC CD4 did improve CD4 monitoring: compared with the standard of care arm, 37% more women in the intervention arm had a CD4 test at antenatal booking and 35% more women had a repeat CD4 test; more women in the POC arm also understood the value and purpose of CD4 testing. Although behavioral change theory supported the idea of knowledge and counseling influencing behavior, either the intervention was not adequately delivered or too many other contextual or socioeconomic factors in individual women's lives diminished these effects and impeded retention in care. An innovative analysis of medication possession ratio found that younger women, those newly diagnosed with HIV or presenting to antenatal care in their third trimester, were more likely to drop from care or be nonadherent 360 days after ART initiation.

## THE VALUE OF RESEARCH REPORTING NO EFFECT

Although some of the studies reported no effect of interventions on rates of retention in care, they still represent important findings. Do they mean that investment in health systems' interventions such as quality improvement is futile or that integration of services does not represent an option for increasing coverage of essential MNCH interventions? Clearly not.

These individual project teams may have been overly ambitious but cannot be criticized for lack of effort, rigor, or commitment. Their study designs may not have fully addressed confounding factors or been powered to detect smaller changes. Improving health systems at scale is likely to take more time and capacity than is generally available to research teams. In addition to the innovative and flexible ground-up approaches exemplified by the project teams, leadership and motivated human resources remain critical ingredients for successful delivery of services.

## OTHER LESSONS FROM INSPIRE

INSPIRE highlighted several other issues from which future research and health programs can learn:

- The 6 studies all adopted different definitions of retention in care and thus makes comparisons difficult. Definitions in 5 studies combined the requirement of attendance at a specific time point with attendance at a minimum number of earlier appointments. One study defined retention as “alive and on ART without any gap in care >60 days after a scheduled visit.” Applying more stringent definitions will mean lower reported rates of retention in care. More critically, research teams struggled to design data collection systems and analytical approaches to describe patterns of attendance according to their a priori definitions. Standardized definitions and analytical approaches that reflect patterns of attendance and ART adherence that translates into health outcomes would serve future research and national HIV programs well;
- Health data collection systems that do not use electronic records are not able to link clinic attendances in different geographic locations or at different time points. Investing in electronic systems at scale would greatly improve our understanding of health-seeking behavior and facilitate the design of public health interventions to support the most vulnerable populations;
- Functional appointment systems, by which health workers could easily review and identify women who had not attended clinic and received ART, were commonly missing. Even in the absence of electronic systems, simple paper-based systems could still be rapidly implemented to address this gap;
- Some populations are more likely to be lost from care than others, eg, younger women and those just initiating ART. Losses may also be greater at different times in the sequence of care, eg, antenatal more than postnatal. Interventions could be directed specifically at these populations and vulnerable periods;
- Sample size estimates for all studies were complicated by the lack of previous relevant published data. Retention rates and intra cluster coefficients calculated during the analyses and provided in these articles will assist sample size calculations for future studies;
- The variation of definitions used by programs and research for “retention in care” and the lack of robust correlates between patterns of attendance and clinical outcomes has important implications for modeling exercises, eg, global estimates of postnatal infant and child HIV infections by the UNAIDS modelling tool SPECTRUM. Less stringent definitions that equate attendance at a single time point, eg, 12 months postpartum with full adherence to ART, are likely to underestimate true retention rates, mother-to-child transmission rates, and, subsequently, the number of children in need of ART.
- The investment and efforts needed to train and support health care workers to provide quality health services in PMTCT are sorely underestimated. The support required to build capacity and confidence of health care workers was essential for successful study implementation. The same investment is needed for delivering consistent quality health services.

## CONCLUSIONS

The past 10 years have seen remarkable developments in knowledge and treatment options for people living with HIV.^14^ The debate is no longer whether ART should be started among everyone who is infected with HIV but how to deliver and sustain treatment at scale including the most vulnerable and difficult to reach populations. Similar challenges are present for other highly effective interventions that enable mothers and children to survive and thrive.

The need for relevant, rigorous, and generalizable evidence from IR has never been greater to complement that which has been learned from programs over the last decade, and to see these findings incorporated.^15^ HIV/AIDS and MNCH programs need innovative, adaptable, cost-effective solutions to realize the ambitious target of the UNAIDS Fast-Track Strategy and the UN Global Strategy for Women's, Children's and Adolescent's Health (2016–2030).^1,16^ WHO fully recognizes the value and importance of IR to provide insights and options that will inform future global policy and recommendations.
